# Advanced Photocatalysts Based on Reduced Nanographene Oxide–TiO_2_ Photonic Crystal Films

**DOI:** 10.3390/ma12162518

**Published:** 2019-08-07

**Authors:** Angeliki Diamantopoulou, Elias Sakellis, Spiros Gardelis, Dimitra Tsoutsou, Spyridon Glenis, Nikolaos Boukos, Athanasios Dimoulas, Vlassis Likodimos

**Affiliations:** 1 Section of Solid State Physics, Department of Physics, National and Kapodistrian University of Athens, Panepistimiopolis, 15784 Ilissia, Greece; 2 Institute of Nanoscience and Nanotechnology, National Center for Scientific Research “Demokritos”, 15310 Agia Paraskevi, Greece

**Keywords:** TiO_2_, photonic crystals, graphene oxide nanocolloids, reduced graphene oxide, photocatalysis

## Abstract

Surface functionalization of TiO_2_ inverse opals by graphene oxide nanocolloids (nanoGO) presents a promising modification for the development of advanced photocatalysts that combine slow photon-assisted light harvesting, surface area, and mass transport of macroporous photonic structures with the enhanced adsorption capability, surface reactivity, and charge separation of GO nanosheets. In this work, post-thermal reduction of nanoGO–TiO_2_ inverse opals was investigated in order to explore the role of interfacial electron transfer vs. pollutant adsorption and improve their photocatalytic activity. Photonic band gap-engineered TiO_2_ inverse opals were fabricated by the coassembly technique and were functionalized by GO nanosheets and reduced under He at 200 and 500 °C. Comparative performance evaluation of the nanoGO–TiO_2_ films on methylene blue photodegradation under UV-VIS and visible light showed that thermal reduction at 200 °C, in synergy with slow photon effects, improved the photocatalytic reaction rate despite the loss of nanoGO and oxygen functional groups, pointing to enhanced charge separation. This was further supported by photoluminescence spectroscopy and salicylic acid UV-VIS photodegradation, where, in the absence of photonic effects, the photocatalytic activity increased, confirming that fine-tuning of interfacial coupling between TiO_2_ and reduced nanoGO is a key factor for the development of highly efficient photocatalytic films.

## 1. Introduction

Over the last decades, TiO_2_ has been widely studied as a low cost, nontoxic, and highly stable photocatalyst, which can degrade a great number of gaseous and aqueous pollutants for environmental remediation [1]. Despite its unique advantages, the photocatalytic efficiency of TiO_2_ is, however, limited by the recombination of photogenerated charge carriers [2] and its wide energy band gap (3.0–3.2 eV), which requires ultraviolet light for electron excitation [3]. Extensive research has accordingly been focused on tailoring titania’s structural and morphological characteristics as well as its compositional and electronic properties by doping, defect engineering, and heterostructuring in order to enhance charge separation and visible-light harvesting [1]. Particular interest has been recently placed on TiO_2_ photonic crystals, a promising titania modification featuring a periodic and mesoporous structure that can manipulate light propagation at specific wavelengths [4]. Because of their periodicity, photonic crystals—the most common being the inverse opal structure fabricated by the self-assembly of colloidal opal templates [5,6]—exhibit photonic band gaps (PBGs), which constitute frequency regions within the crystal where electromagnetic irradiation cannot propagate. At wavelengths near the PBG edges, slow photon effects appear, i.e., light propagation at reduced group velocity, leading to an increase in the optical path length and an enhancement of light–matter interactions [7,8,9,10].

Among the diverse heterostructured TiO_2_ photocatalysts, nanocomposites comprising titania with graphene or its derivatives have emerged as a distinct material class of topical interest because of graphene materials’ exceptional electron acceptor action and charge transport, together with their unique two-dimensional morphology and broadband light absorption [11,12,13]. In particular, graphene oxide (GO) has attracted much attention since 2004, when graphene research first emerged [14], as a solution-processable graphene precursor. It consists of graphene sheets with randomly distributed oxygenated groups on each plane and thus contains both sp^2^ and sp^3^ carbon atoms [15]. Owing to its abundant oxygen groups, GO demonstrates rich surface chemistry and reactivity, which can be favorably exploited for the assembly of highly efficient GO–TiO_2_ composite photocatalysts with strong interfacial coupling and improved charge separation under optimal conditions [16,17] as well as exceptional pollutant adsorption ability [18]. Significant efforts have been recently devoted to determine and control the size and thickness variation of micrometer-sized GO sheets [19], which are crucial for their surfactant properties and emulsion ability [20,21] as well as for efficient GO incorporation and electrical performance as filler in polymer composites [22]. In the case of composite photocatalysts, GO nanosheets may act as scavenger and shuttle of TiO_2_-photogenerated electrons, inhibiting electron-hole recombination depending on the oxidation state of GO and the nature of the pollutant [23,24,25,26], although GO may also operate as a sensitizer of TiO_2_ via the formation of p–n junctions that enable electron transfer from GO to TiO_2_ under visible light [27], depending on the variation of the π–π* localized states of the inhomogenously distributed aromatic sp^2^ domains, especially in reduced GO (rGO) [23,28]. Moreover, electrodeposition of GO coatings on anodized TiO_2_ nanotube arrays and post-thermal reduction was recently shown to enhance the photocatalytic performance of pristine GO–TiO_2_ films due to the enhanced charge separation and adsorption ability of the GO sheets [29]. Controlled synthesis of stable aqueous GO nanocolloids consisting of nanometer-sized GO nanosheets (nanoGO) was recently demonstrated by the chemical exfoliation of graphite nanofibers [30]. Compared to regular GO sheets, which extend over several microns, the smaller size of nanoGO sheets increases the oxygen-containing functional groups in the basal planes and the sheet edges, leading to higher colloidal stability and hydrophilicity as well as more available surface reaction and anchoring sites and enhanced interfacial coupling with TiO_2_ nanomaterials [31]. Very recently, surface functionalization of PBG-engineered TiO_2_ photonic films by GO nanocolloids was demonstrated as an effective approach to enhance the photocatalytic degradation of methylene blue (MB) dye under UV-VIS and visible light by combining the advantages of macroporous inverse opals of improved light harvesting, surface area, and mass transport with the high adsorption capacity, reactivity, and charge separation of GO nanocolloids [32]. In the case of UV-VIS irradiation, nanoGO deposition on the titania inverse opals led to a marked improvement of the MB photocatalytic degradation rate, which was related to the synergy of slow photon amplification upon spectral overlap of the low-energy edge of the incomplete TiO_2_ inverse opal PBG (stop band) with the dye electronic absorption, enhanced MB adsorption due to the electrostatic interactions of surface oxygen groups and π–π coupling with the dye molecules [33], and interfacial nanoGO–TiO_2_ charge transfer [32]. Likewise, a distinct acceleration of dye photodegradation kinetics was observed for the nanoGO–TiO_2_ photonic films under visible light, which was related to the combination of increased dye adsorption with the slow photon-intensified dye photocatalysis based on the self-sensitization mechanism. 

In this work, postreduction of PBG-engineered graphene oxide–titania photonic films was explored in order to elucidate the interplay of interfacial electron transfer with pollutant adsorption via GO’s surface functional groups and thus provide a means to further improve the photocatalytic performance of nanoGO–TiO_2_ photonic crystals. To this end, well-ordered TiO_2_ inverse opal films fabricated by the coassembly technique with tuned stop band for the optimal slow photon-assisted MB photodegradation were functionalized by GO nanosheets and reduced by thermal annealing in He at different temperatures (200 and 500 °C). Thermal reduction of nanoGO is expected to increase the sp^2^/sp^3^ ratio of carbon atoms, leading to a decrease in the energy gap and an increase in the conductivity of rGO nanosheets [34,35]. Comparative performance evaluation of the pristine and reduced nanoGO–TiO_2_ inverse opals was performed on MB dye degradation under UV-VIS and visible light as well as UV-VIS photodegradation of colorless salicylic acid (SA), an emerging water pollutant rarely investigated by photonic crystal photocatalysts [4]. Despite the decrease in the GO amount and the reduction in surface oxygen groups on the post-treated photonic films, a clear enhancement of the photocatalytic activity was identified, indicating that optimization of charge transfer between reduced GO and the nanocrystalline TiO_2_ inverse opal walls may effectively alleviate electron-hole recombination and promote their photocatalytic performance.

## 2. Materials and Methods

### 2.1. Materials

Monodisperse polystyrene (PS) spheres with diameter of 425 nm were purchased from Microparticles GmbH (Berlin, Germany) in the form of colloidal dispersion of 5% solids (w/v) in deionized (DI) water (2.3% CV, SD = 0.01 μm). The specific sphere diameter was selected because the corresponding nanoGO–TiO_2_ inverse opals presented the optimal photocatalytic activity on MB degradation in comparison to the ones synthesized by colloidal PS spheres of other diameters (220, 350, and 510 nm) [32]. Titanium(IV) bis(ammonium lactato)dihydroxide (TiBALDH) 50 wt.% aqueous solution (388165, Sigma-Aldrich, St. Louis, MO, USA), graphene oxide aqueous nanocolloidal dispersion consisting of single-layer GO sheets down to a few nanometers in lateral width, 2 mg/mL in H_2_O, 42.0%–52.0% C in dry basis (795534, Sigma-Aldrich, St. Louis, MO, USA), and Hellmanex^™^ III were obtained from Sigma-Aldrich. All other reagents were of analytical or ACS reagent grade: ethanol (EtOH, 32221, Honeywell Riedel-de Haën, puriss. p.a., absolute, ≥99.8%), methanol (MeOH 179337, Sigma-Aldrich, ACS reagent, ≥99.8%), acetone (32201, Honeywell Riedel-de Haën, ACS reagent, ≥99.5%), hydrochloric acid (HCL, 30721 Fluka, ACS reagent, fuming, ≥37%). 

### 2.2. Inverse Opal Fabrication and Surface Modification

Titania inverse opal films were fabricated via the evaporative coassembly of the PS colloidal spheres with the hydrolyzed TiBALDH titania precursor [36]. This synthesis route was selected over the more common successive deposition method, which involves liquid infiltration and subsequent removal of the polymeric opals, as it produces high-quality inverse opal films with large photonic domains [32]. In particular, cleaned glass substrates (S8902 Sigma-Aldrich) by Hellmanex™ III andultrasound acetone–EtOH washing) were nearly vertically suspended into glass vials, each containing 8 mL of 0.2 wt.% PS dilute sphere suspension and 0.168 mL of titania precursor, synthesized by stirring of 1.23 mL TiBALDH solution, 1.5 mL HCl 0.1 Mand 2.85 mL EtOH for 1 h. The vials were placed in a heating oven at 55 °C until the solvent fully evaporated over 3 days, leading to the deposition of a thin film on the glass substrates. The obtained films were calcined at 500 °C in air for 2 h, resulting in the removal of the polymer matrix and crystallization of titania in the inverse opal structure. The fabricated TiO_2_ photonic crystals were designated as PC. The surface functionalization of the inverse opals was performed by dipping the films for 24 h in the nanocolloidal GO dispersion, whose pH had been stabilized at 10 by periodically adding 1–2 drops of NaOH aqueous solution (1 M) and intermediate 10 min stirring. The modified photonic films were denoted as GOnano-PC. The reduction was carried out thermally by calcinating the GOnano-PC films for 2 h under He flow at 200 and 500 °C, producing the rGO-modified photonic crystals named as rGOnano(200)-PC and rGOnano(500)-PC, respectively.

### 2.3. Material Characterization

The morphology and phase composition of the inverse opals was studied using scanning electron microscopy (SEM, Quanta Inspect, FEI, Eindhoven, The Netherlands) coupled with energy-dispersive X-ray spectroscopy (EDXDX4, EDAX, Mahwah, USA, and transmission electron microscopy (TEM, CM20, FEI, Eindhoven, The Netherlands) augmented by energy-filtered TEM (GIF 200, Gatan, Pleasanton, CA, USA). The composition of the films was analyzed by X-ray photoelectron spectroscopy (XPS, SPECS, Berlin, Germany). Core-level photoemission spectra were collected with a PHOIBOS 100 (SPECS, Berlin, Germany) hemispherical analyzer at a pass energy of 15 eV utilizing Mg Kα radiation at 1253.6 eV. The binding energy scale was calibrated using the position of both Au 4f_7/2_ and Ag3_d5/2_ peaks at 84 and 368.3 eV, respectively, measured on clean gold and silver foils. Gaussian–Lorentzian shapes (Voigt functions) were used for deconvolution of the recorded spectra after standard Shirley background subtraction. The structural properties of the films were investigated by micro-Raman spectroscopy (inVia Reflex, Renishaw, UK) with 514.5 nm excitation from an Ar^+^ ion laser. The laser beam was focused on the sample by means of a ×50 (ΝA = 0.75) objective under low power density (0.05 mW/μm^2^) to avoid local heating. The optical properties of the photonic films were determined by diffuse and specular reflectance UV-vis spectroscopy (Cary 60 UV-Vis, Agilent, USA) equipped with a fiber optic diffuse reflectance accessory (Barrelino) and a 15° specular reflectance one (PIKE, UV-VIS 15Spec), using a Halon standard and a UV Al mirror for baseline measurements, respectively. Charge transfer was investigated by photoluminescence (PL) measurements performed by lock-in amplification techniques. The excitation beam was generated by a light-emitting diode at 275 nm and modulated by a mechanical chopper (SR540, Stanford Research Systems, Sunnyvale, USA). The modulated PL signal was analyzed by a 1/4 monochromator (77200, Oriel, Irvine, USA) and detected by a Si photodiode (FDS1010, Thorlabs GmbH, Munich, Germany) using a benchtop photodiode amplifier ( PDA200C, Thorlabs GmbH, Munich, Germany) and a lock-in amplifier (PAR 126, Princeton Applied Research, Oak Ridge, TN, USA).

### 2.4. Photocatalytic Performance

The photocatalytic activity of the inverse opals was evaluated on the aqueous phase degradation of methylene blue [3,7-bis(dimethylamino)phenazathionium chloride] (MB, A18174, Alfa Aesar) and salicylic acid (SA, 247588, Sigma-Aldrich) under UV-VIS and visible light. The photonic films (~1.5 cm^2^) were placed horizontally at the bottom of beakers containing aqueous MB (4 mL, 3 μM) or SA (3 mL, 25 μΜ) solutions, where they were left for 60 min under dark conditions to reach adsorption–desorption equilibrium under stirring [32,37]. To further enhance the adsorption of salicylic acid on the titania films, the pH of the SA solution had been stabilized at 3 using dilute HCL solution [37]. The illumination source was a 150 W Xe lamp (6255, ORIEL GmbH, Darmstadt, Germany), and UV-VIS irradiation was selected by the combination of a long-pass edge filter with cut-on wavelength of 305 nm (20CGA-305, Newport) and a heat reflective mirror (Newport 20CLVS-3 CoolView™, T_avg_ = 85% at 332–807 nm, R_avg_ = 95% at 840–1500 nm, 20CLVS-3 CoolView™, Newport, Irvine, USA). Visible-light irradiation was selected by an additional long-pass edge filter with cut-on at 400 nm (20CGA-400, Newport, Irvine, USA). The horizontal Xe beam was directed on the film surface via a UV-enhanced Al mirror (Newport ValuMax 20D520AL.2, λ/10, R_avg_ > 90% at 250–600 nm, ValuMax 20D520AL.2, Newport, Irvine, USA). The power density of the incident beam reaching the film surface was 2.8 mW/cm^2^ in the case of MB degradation and nearly 1 sun (96 mW/cm^2^) for the SA in the UV-VIS spectral range. At given time intervals, a small (0.5 mL) aliquot of the MB/SA solution was withdrawn and quantitatively analyzed using a 10 mm path length quartz micro cell (105B-QS, 500 μL, HELMA Analytics) in the Cary 60 spectrophotometer. Subsequently, the analyzed aliquot was poured back into the reacting solution and the illumination continued. The photocatalytic experiments were performed in triplicate, and standard errors were calculated for the mean kinetic constants. The stability of the photocatalytic films was tested by recycling three times the SA degradation test for the same film, with intermediate cleaning of SA residues on the used film by 1 h UV-VIS illumination in 4 mL deionized water.

## 3. Results and Discussion

### 3.1. Morphology, Structural, and Optical Properties

**Figure 1:**
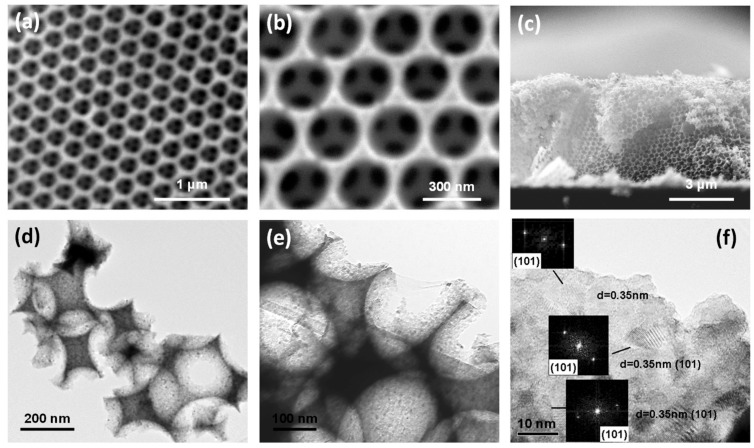
(**a**)–(**c**) Top view and cross-section SEM and (**d**)–(**f**) TEM images of the rGOnano(200)-PC inverse opal at different magnifications. The insets in the high-resolution TEM (HR-TEM) image (**f**) show the fast Fourier transform (FFT) patterns of the indicated areas.

The morphology of the TiO_2_ photonic crystals was examined by SEM and TEM. Figure 1a–c displays representative SEM images of the rGOnano(200)-PC inverse opal featuring a well-ordered, periodic macroporous structure corresponding to the (111) planes of an fcc lattice. The spherical macropores were hexagonally arranged and were well interconnected through smaller pores with diameter of ca. 50–90 nm. The average diameter of the macropores was 245(10) nm, a value much smaller than that of the original PS colloidal spheres, namely 425 nm, confirming the persistent shrinkage of metal oxide inverse opals by means of the amorphous-to-crystalline phase transition and the inorganic matrix volume decrease after calcination [38]. According to cross-section SEM, shown in Figure 1c, the film thickness was around 4.5 μm. Furthermore, it was observed that surface modification of the TiO_2_ inverse opals by nanoGO as well as their subsequent thermal reduction at 200 and 500 °C left both the morphology and the size of the macropores pores intact, identical to those of the pristine photonic films [32]. 

**Figure 2:**
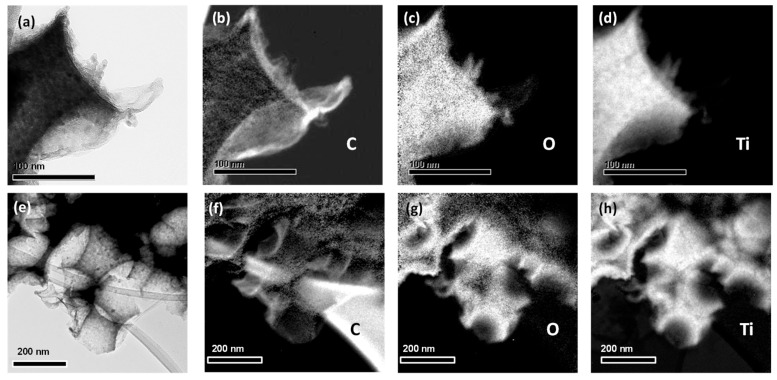
Bright-field TEM image and the corresponding C, O, and Ti energy-filtered TEM (EF-TEM) elemental maps of (**a**)–(**d**) GOnano-PC and (**e**)–(**h**) rGOnano(200)-PC.

TEM images at successively higher magnifications, displayed in Figure 1d–f, show that the walls of the inverse opal skeleton were mesoporous, consisting of ca. 10 nm titania nanoparticles with the most common 0.35 nm *d*-spacing arising from the (101) planes of the anatase TiO_2_ phase. To verify the deposition of graphene oxide and reduced graphene oxide sheets on the inverse opals, elemental mapping was performed by energy-filtered (EF) TEM. Figure 2a–h show a bright-field TEM image of the inverse opal skeleton and the corresponding C, O, and Ti EF-TEM elemental maps for the GOnano-PC and rGOnano(200)-PC films, respectively. The presence of carbon, besides Ti and O, on the nanocrystalline TiO_2_ walls of a void macropore could be identified in both cases, verifying the successful surface modification of the titania photonic films with GO nanosheets.

Due to their reduced lateral size, GO nanosheets could be fully incorporated in the inverse opal macropores, justifying the absence of any noticeable effects on the periodicity and macropore diameter of the photonic crystals. On the other hand, surface functionalization of the TiO_2_ inverse opals by regular, micrometer-sized GO sheets resulted in extended coverage of the nanocrystalline TiO_2_ walls by GO sheets over several macropores [32]. The extensive GO coverage of the TiO_2_ skeleton can impede the pore accessibility to the diffusing pollutant molecules and thus impose an adverse effect to the photocatalytic process, similar to the partial surface clogging observed for benchmark mesoporous P25 films subjected to the same functionalization treatment [32].

**Figure 3:**
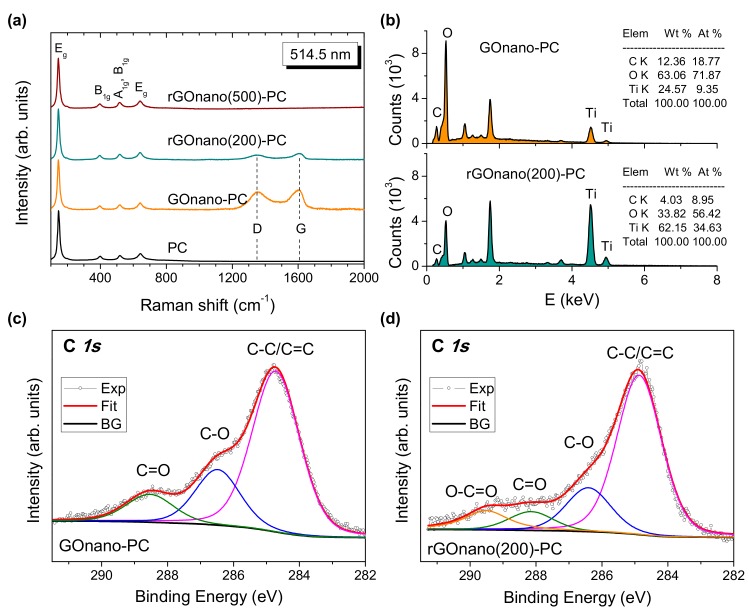
(**a**) Raman spectra of the pristine and modified inverse opals. (**b**) Energy-dispersive X-ray spectroscopy (EDX) spectra and C, O, Ti elemental analysis for GOnano-PC and reduced rGOnano(200)-PC. C *1s* X-ray photoelectron spectroscopy (XPS) spectra for the (**c**) GOnano-PC and (**d**) rGOnano(200)-PC films.

The structural properties of the TiO_2_ photonic crystals and their functionalization with GO were further investigated by Raman spectroscopy. Figure 3a compares the Raman spectra of the pristine and modified photonic films at 514.5 nm. All inverse opals exhibited the characteristic bands of anatase TiO_2_ at approximately 148 (E_g_), 398 (B_1g_), 520 (A_1g_ + B_1g_), and 643 (E_g_) cm^−1^. No traces of polystyrene, rutile, or brookite could be detected, confirming the removal of the polymer matrix as well as the crystallization of titania in the anatase phase after thermal treatment [39]. Furthermore, appreciable broadening and shift of the anatase Raman peaks was observed, especially for the low-frequency E_g_ mode, which shifted to 148 cm^−1^ and broadened to full-width at half-maximum of 18 cm^−1^, indicative of the formation of anatase nanocrystals with size ≤10 nm [40], in close agreement with the direct imaging of the nanocrystalline inverse opal walls by HR-TEM (Figure 1).

In addition to the anatase Raman bands, the GOnano-PC film presented the characteristic G-mode of graphene oxide, stemming from the stretching of sp^2^ carbon atoms, and the highly dispersive D-band, activated by defects in GO in the frequency range of 1000–2000 cm^−1^. The G-band displayed a distinct asymmetric lineshape with spectral weight being shifted at higher frequency [24], related to the diverse contributions of alternating single–double carbon bonds [41]. Reduction at 200 °C led to a significant decrease in the intensity of the G- and D-modes relative to that of anatase by nearly 65%, suggesting that a considerable amount of GO is removed from the film when it is thermally treated [30]. This behavior could be clearly identified in the Raman spectrum of rGOnano(500)-PC, where the G- and D-peaks could be hardly detected, indicating that the film almost recovered its pristine state after annealing at 500 °C. In addition, the intensity ratio I_D_/I_G_ determined from the integrated areas of the D- and G-bands, which is the characteristic probe of the sp^2^ domain size as well as the defect density in graphitic materials [42,43], decreased from the value of 1.60 for GOnano-PC to 1.54 for rGOnano(200)-PC films. The partial restoration of sp^2^ bonds and the decrease of defects in the GO nanosheets could be accordingly inferred [44,45,46], considering that GO thermal reduction does not cause a large expansion of the average graphitic domain size [47]. The loss of GO sheets was further supported by EDX, as shown in Figure 3b, where a nearly 70% decrease in the C content was observed for rGOnano(200)-PC with respect to the GOnano-PC. The surface composition of the nanoGO–TiO_2_ inverse opals was investigated by XPS. Figure 3c,d compares the deconvoluted XPS C1s spectra of the GOnano-PC and rGOnano(200)-PC films. Both spectra were highly functionalized with oxygenated groups such as epoxy/hydroxyl groups (C–O at ~286.5 eV), carbonyl group (C=O at ~288.3 eV), and carboxyl group (O–C=O at ~290 eV) [24,47]. As expected, increased amounts of oxygenated groups were present in GOnano-PC compared to the thermally reduced film. It was found out that, for the nanoGO-PC, the ratio between the strongest C *1s* peak, corresponding to the signal arising from C atoms in the C–C/C=C configurations and the other components corresponding to the oxygenated groups, was 1.5, indicating a considerable oxidation degree. After thermal reduction, this ratio reached a value of 2.1, indicative of the loss of surface oxygen groups and the enhancement of the sp^2^ carbon bonds [47].

**Figure 4:**
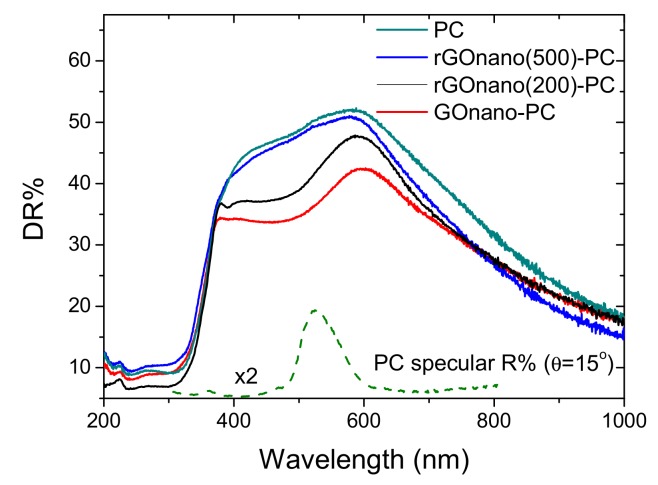
UV-VIS diffuse reflectance (DR%) spectra of the pristine and modified photonic crystals. The dashed line shows the specular R% spectrum of the pristine PC film at 15° incident angle.

The decrease in the GO amount on the TiO_2_ inverse opals upon thermal reduction was further evidenced in the diffuse reflectance (DR%) spectra, as shown in Figure 4. Besides the anatase band gap edge below 400 nm, all films presented a broad DR% peak at approximately 580 nm, close to the low energy (red) edge of the stop band identified at 525 nm in the pristine PC by the 15° incidence specular (R%) spectra. It should be noted that the DR% intensity considerably exceeded the specular one due to the presence of disorder with respect to the relatively coarse beam size (1.5 mm^2^) that probes film areas comprising several domains of variable thickness and surface flatness [32]. Diffuse reflectance was more intense at higher wavelengths than the stop band predicted by the R% spectra because slow photons at the “red” stop band edge, which are localized at the high dielectric medium, i.e., the titania skeleton, experience a longer optical path and are thus more likely to be scattered. Surface modification of the titania photonic films with nanoGO as well as the post-thermal treatment hardly affected the position of the broad DR% photonic peak, reflecting the weak effect of the GO nanosheets on the inverse opal macropores and periodicity, in agreement with SEM analysis (Figure 1). However, a distinct decrease in the initial DR% was observed for GOnano-PC, arising from the broadband absorption of the GO nanosheets [30]. On the other hand, the DR% reflectance was gradually restored after post-treatment at 200 and 500 °C, corroborating the corresponding GO losses on the thermally reduced photonic films.

The stop band spectral position can be approximated by modified Bragg’s law for first-order diffraction from the (111) planes [4]:

$$\lambda =2d_{111}\sqrt{n_{eff}^{2}-\sin^{2}\theta }$$

where *λ* is the stop band wavelength; $d_{111}=\sqrt{2/3}D$ is the interplanar spacing of the (111) planes, with *D* being the macropore diameter; and *n_eff_* is the volume-weighted average of the void spheres’ refractive index $n_{\mathrm{void}}$ and titania $n_{{\mathrm{TiO}}_{2}}$ occupying the inverse opal skeleton, defined by $n_{eff}^{2}=n_{\mathrm{void}}^{2}f+n_{{\mathrm{TiO}}_{2}}^{2}\left(1-f\right)$, with *f* being the filling fraction (*f* = 0.74 for the ideal *fcc* lattice) and *θ* being the angle between the incident beam and the plane normal. Using *θ* = 15° together with the measured stop band position and macropore diameter for $n_{{\mathrm{TiO}}_{2}}=2.55$ and $n_{\mathrm{air}}=1.0$, the *n*_eff_ value of 1.34 and titania skeleton filling fractions (1 − *f*) of 0.14 were determined in air, in perfect agreement with previous results [32]. Moreover, using the obtained TiO_2_ filling fraction and $n_{H_{2}O}=1.33$, the stop band position of 626 nm was calculated for the inverse opal films in aqueous medium, where the photocatalytic reaction takes place.

### 3.2. Photocatalysis

**Figure 5:**
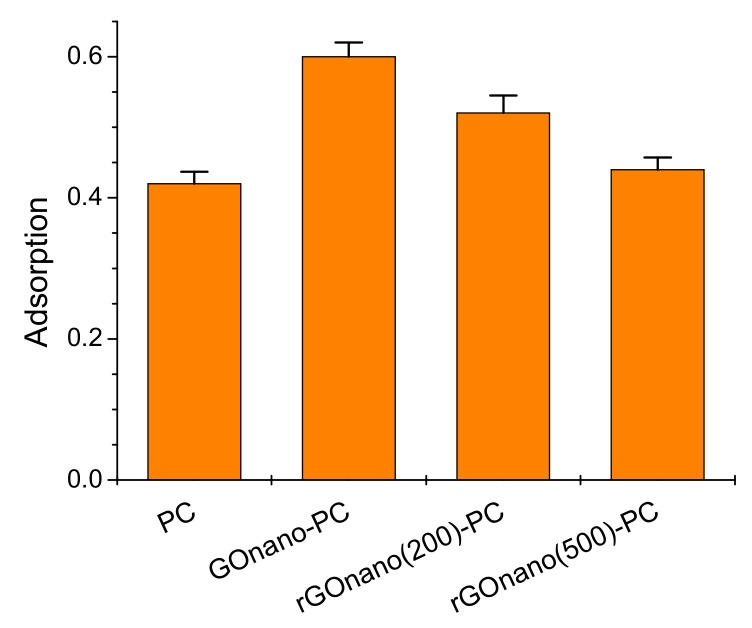
Methylene blue (MB) adsorption on the pristine, GO- and rGO-modified TiO_2_ photonic crystals under dark conditions.

Figure 5 displays the adsorption of methylene blue on the different inverse opals under dark conditions. A marked rise of MB dark adsorption was observed after nanoGO functionalization, which can be attributed to the electrostatic interactions between the negatively charged oxygenated groups of GO and the positively charged amino groups of the cationic MB dye. These interactions can be further assisted through the π–π coupling of GO itinerant sp^2^ electrons with delocalized electrons in the aromatic rings of the MB molecules [33]. Dark adsorption lessened with thermal reduction, reflecting the decrease in surface oxygen groups as well as the GO amount on the photonic films, as inferred from the Raman, EDX, XPS, and UV-VIS results. In fact, the MB adsorption of the rGOnano(500)-PC film was only slightly higher than that of the pristine one, indicating the defunctionalization of the sample after thermal annealing at 500 °C. The small difference in MB adsorption between the pristine and the rGOnano(500)-PC photonic film can be related to residual rGO that could not be detected by Raman spectroscopy, but it enhanced the adsorption slightly.

**Figure 6:**
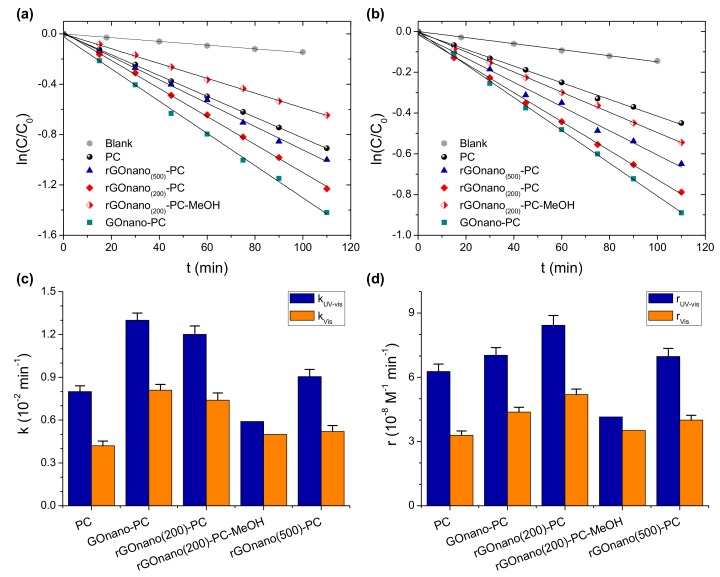
MB photodegradation kinetics of the inverse opals under (**a**) UV-VIS and (**b**) VIS irradiation. (**c**) Apparent rate constants k and (**d**) reaction rates r = kC_0_ under UV-VIS and visible light.

Figure 6a,b presents the MB photodegradation kinetics for the pristine, GOnano and rGOnano photonic crystals under UV-VIS and VIS irradiation, respectively. In all cases, the ln(C/C_0_) = f(t) plots were linear, indicating that the MB photodegradation followed first-order kinetics. Figure 6c summarizes the apparent rate constants k_UV-VIS_ and k_VIS_ calculated from the slopes of the corresponding plots. It was evident that the GO-functionalized film presented the highest apparent rate under both UV-VIS and VIS illumination, implying that deposition of GO nanosheets on the titania films can boost their photocatalytic performance. This behavior can be attributed not only to the higher MB adsorption but also to the enhanced charge separation induced by GO as well as the slow photon amplification produced by the specific photonic crystal [32]. In particular, photogenerated electrons in TiO_2_ by UV-VIS light can be scavenged and shuttled in the GO nanosheets, leading to the decrease in electron-hole recombination. As a result, MB molecules could be degraded not only by hydroxyl radicals formed by valence band holes of TiO_2_ but also via intermediate peroxide or other radicals formed in the GO sheets [18]. Nevertheless, photocatalytic experiments in the presence of 0.01 M methanol (MeOH) as hydroxyl radical scavenger [48] showed roughly 50% and 30% reduction of the corresponding k_UV-VIS_ and k_VIS_ constants, respectively, as shown in Figure 3 for the rGOnano(200)-PC, verifying the dominant role of ^•^OH radicals in MB degradation, especially under UV-VIS light [49]. In addition, the PC film fabricated by the 425 nm PS spheres presented the optimal photocatalytic performance due to slow photon effects [32]. In the present case, the red edge of the 626 nm stop band for the PC film in water is also very close to the MB electronic absorption at 664 nm, where multiple scattering due to “red” slow photons localized in the titania skeleton occur, leading to the acceleration of MB photodegradation kinetics. Furthermore, GO can promote the self-sensitized photocatalytic oxidation of MB molecules, which is the main degradation mechanism under visible light, where TiO_2_-photogenerated carriers are absent due to the wide anatase band gap. In this case, the strong MB adsorption on the GO nanosheets is crucial for the interfacial electron transfer and the enhanced photodegradation as the electrons of the adsorbed MB molecules excited by visible light are scavenged by GO–TiO_2_, forming the oxidation radicals that degrade the pollutant.

Nevertheless, it can be noticed that the initial concentration C_0_ of the MB solution in the photocatalytic experiments was not the same for all samples because of the different degree of MB adsorption on each film (Figure 5). To take the C_0_ variation into account, the reaction rate r was estimated, which, for low (<mM) MB concentrations, is proportional to C_0_, according to the Langmuir–Hinshelwood (L–H) model, r = kC_0_ [49]. Figure 6d compares the reaction rates for all inverse opals under UV-VIS and visible illumination. In both cases, the maximum r value was observed for the rGOnano(200)-PC and not for the GOnano-PC film despite the high apparent constant k of the latter, indicating that the photocatalytic activity of the GO-modified sample improved after reduction at 200 °C. This behavior would further suggest enhanced charge transfer and transport on the rGO nanosheets due to their higher conductivity and favorable work function [23,35]. It appears that, in the case of the rGOnano(200)-PC film, the enhancement of electron transfer/transport is the definitive factor in its photocatalytic efficiency as MB adsorption was lower than that for the GO-functionalized sample. On the other hand, the reaction rate of GOnano-PC decreased when the film was thermally reduced at 500 °C, with its value being almost the same as that for the pristine one. This behavior complies favorably with previous results on the fabrication of highly efficient GO–TiO_2_ powder nanocomposites for the photocatalytic decomposition of different water pollutants [16,24,50], where a mild thermal reduction post-treatment at 200 °C in N_2_ atmosphere was found to enhance the optimal assembly and photocatalytic activity of TiO_2_ nanoparticles on GO sheets. Although direct comparison of the films’ photocatalytic performance with literature values is rather difficult due to differences in dye concentration, film size and mass, and irradiation density, the present rates are comparable or even higher than the r~10^−8^ M^−1^ min^−1^ that can be derived from the UV-VIS MB degradation k values reported in a thorough study of TiO_2_ inverse opals (5 ppm MB with 2 mg and 5 cm^2^ area of 2.5–3 μm thick photonic films under simulated solar light) [51] and most importantly exceed those of the benchmark mesoporous P25 films under the same conditions [32].

**Figure 7:**
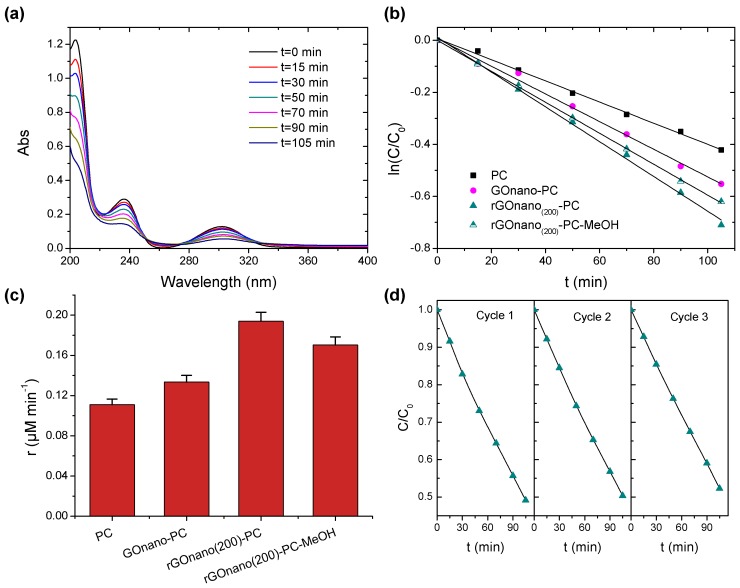
(**a**) Salicylic acid (SA) absorbance variation for the rGOnano(200)-PC film under UV–VIS light. Photodegradation (**b**) kinetics and (**c**) reaction rates of SA for the PC, GOnano-PC and rGOnano(200)-PC inverse opals under UV-VIS irradiation. (**d**) SA photodegradation kinetics after three successive photocatalytic tests using the rGOnano(200)-PC film.

To explore charge separation effects on the GO- and rGO-functionalized TiO_2_ photonic crystals, the photodegradation of salicylic acid under UV-VIS irradiation was subsequently investigated. Salicylic acid is a colorless water pollutant that absorbs in the UV range, as shown in Figure 7a, away from the stop band of the photonic films (626 nm in water), excluding the contribution of slow photon effects that are dominant in the MB dye degradation. 

Moreover, its adsorption on the titania films, although enhanced at pH = 3 due to the SA chemisorption on the TiO_2_ surface [37,52], is relatively weaker compared to that of MB molecules with minimal effects on the corresponding reaction rates [53]. Comparative SA photodegradation experiments for the PC, GOnano-PC and rGOnano(200)-PC films were accordingly exploited as an indirect means to assess the efficiency of charge separation for GO- and rGO-functionalized films. SA degradation followed first-order kinetics, as shown in Figure 7b. The corresponding plot for rGOnano(500)-PC was similar to that of the pristine one and thus not included in the graph. The reaction rates r, depicted in Figure 7c, revealed an improvement of the photocatalytic activity for GOnano-PC, which was further increased after thermal reduction, despite the loss of GO nanosheets and surface oxygen groups. In this case, the improved photocatalytic activity for rGOnano(200)-PC can be mainly attributed to the improved charge separation arising from the enhanced electron transfer from TiO_2_ to the rGO nanosheets due to their lower work function and higher conductivity [23,34,47]. SA photodegradation by rGOnano(200)-PC in the presence of MeOH as ^•^OH scavenger revealed a weak effect on the reaction rate, corroborating the major role of direct SA oxidation by TiO_2_ valence band holes [37]. Moreover, consecutive SA photodegradation tests for the optimal rGOnano(200)-PC photocatalyst, shown in Figure 7d, corroborated the film stability (roughly 6% reduction of the SA degradation was observed after three photocatalytic cycles), similar to the excellent stability of the GOnano–TiO_2_ inverse opals on MB degradation [32]. 

**Figure 8:**
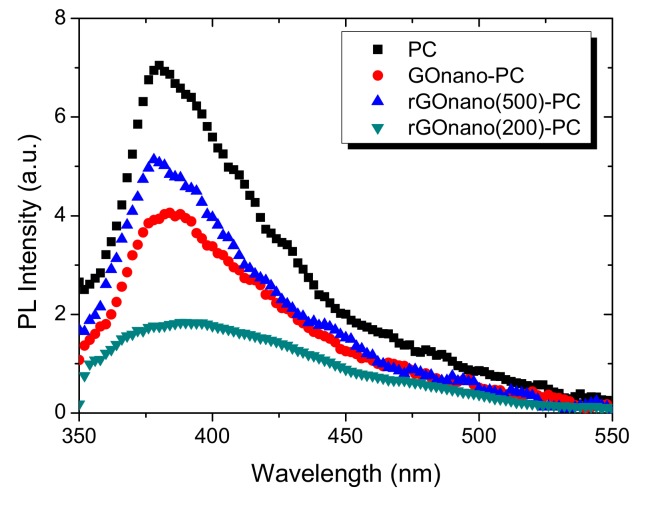
Photoluminescence (PL) spectra of the pristine, GO-functionalized and rGO-functionalized photonic crystals.

The charge separation on the rGO-modified photonic films was further supported by PL spectroscopy (Figure 8). The PL spectra of the pristine film exhibited a broad band at 380 nm, whose intensity was drastically reduced after the surface deposition of GO, verifying the decrease in electron-hole recombination and corroborating the interfacial transfer of UV photogenerated electrons from TiO_2_ to the GO nanosheets. Postreduction of GO at 200 °C led to a further decrease in the PL intensity, reflecting the promotion of electron transfer. On the other hand, PL intensity was augmented after reduction at 500 °C due to the defunctionalization of the film. The PL intensity of the rGOnano(500)-PC, however, did not reach that of the pristine photonic crystal, probably owing to residual graphene oxide on the sample even after its calcination.

## 4. Conclusions

TiO_2_ inverse opals, fabricated via the coassembly of polystyrene colloidal spheres with the water soluble TiBALDH precursor, were surface-functionalized with GO nanosheets and, subsequently, thermally reduced at 200 and 500 °C. Neither GO surface modification nor postreduction affected the highly ordered macroporous structure of the TiO_2_ photonic films, leaving the photonic stop band positions intact. Raman, EDX, and XPS spectroscopies corroborated the successful GO functionalization of the TiO_2_ inverse opals and disclosed that, upon post-thermal treatment, the amount of nanoGO was moderated on the modified films along with the partial recovery of sp^2^ domains. Aqueous phase photodegradation of the MB dye under UV-VIS and visible light showed that thermal reduction of the GO–TiO_2_ photonic films at 200 °C, in synergy with slow photon amplification, improved the MB photocatalytic degradation rate despite the loss of oxygen functional groups and GO nanosheets, indicative of enhanced charge separation due to the lower work function and higher conductivity of the rGO nanosheets. The intensification of interfacial charge transfer was further supported by both photocatalytic experiments under UV-VIS light using salicylic acid as emerging water pollutant and PL spectroscopy. In that case, the SA photocatalytic degradation was drastically increased on the post-treated rGO–TiO_2_ inverse opals despite the absence of photonic effects. This confirms that further optimization of the amount of reduced GO nanosheets and fine-tuning of their interfacial coupling with the TiO_2_ nanoparticles on the nanocrystalline walls of the macroporous inverse opal films is a promising route to alleviate electron-hole recombination and boost their photocatalytic performance.
